# Assessing Telemedicine Demand and Viability in Indonesian Geriatric Clinics: A Comprehensive HOT FIT and Sociotechnical Analysis

**DOI:** 10.2174/0118746098302999240522092726

**Published:** 2024-06-12

**Authors:** Dewi Shinta Kemala Sari, Nur Indrawati Lipoeto, Hafni Bachtiar, Indra Catri, Nina Kemala Sari, Rima Semiarty

**Affiliations:** 1 Faculty of Medicine, Universitas Andalas, Jl. Perintis Kemerdekaan No. 94, Kawasan Kampus Limau Manis, Padang 25129, West Sumatra, Indonesia;; 2 Faculty of Medicine, Universitas Indonesia. Jl. Salemba Raya No. 6, Jakarta Pusat 10430, Indonesia

**Keywords:** Telemedicine, geriatrics, hospital, needs, grounded theory, aging, health service

## Abstract

**Introduction:**

The growing elderly population in Indonesia presents challenges for the healthcare system, prompting the exploration of telemedicine as a solution. However, its effective implementation in Indonesia faces obstacles.

**Methods:**

This research aimed to develop a comprehensive geriatric telemedicine framework in Padang City by studying multiple stakeholders. We employed qualitative methods, including in-depth interviews, across two hospitals, a Health Office, and a Community Health Center, involving 18 elderly participants.

**Results:**

The study identified ten key dimensions for geriatric telemedicine services: technology, Human-Computer Interface (HCI), infrastructure, system workflow, clinical content, people (diverse roles), organization (ecosystem, service workflow, internal and external regulations), and financing (social security agency on health and independent). We used the Human-Organization-Technology Fit and Sociotechnical System approaches for analysis.

**Conclusion:**

The study suggests implications for future implementation and advocates for broader participant involvement, information technology (IT) studies for system development, and longitudinal evaluations to assess the impact on elderly health outcomes.

## 1 INTRODUCTION

The global phenomenon of population aging poses a significant challenge for healthcare systems worldwide, including in Indonesia. Moreover, as the elderly population grows, so does the prevalence of chronic diseases and geriatric syndromes, necessitating more intensive treatment and care [1]. Indonesia has been experiencing this demographic shift since 2021, with the elderly population increasing by at least 3% over the past decade (2010-2021) to reach 10.82% (Statistics Indonesia, 2022). West Sumatra Province, in particular, has a notably high percentage of elderly residents (Indonesian Central Bureau of Statistics, 2022). Surprisingly, despite the rising need for medical care [2], the interest of elderly individuals in seeking treatment at health facilities has been decreasing. A national survey reveals that 42% of older adults report health complaints, and only 15% seek outpatient treatment.

Data from the Central Bureau of Statistics shows that, from 2020-2022, the percentage of elderly individuals who sought outpatient care dropped from 24.6% (in 2020) to 15.69% (in 2022). Instead, many chose to self-medicate, and 2-3% did not seek any treatment, particularly among the elderly and disabled (Indonesian Central Bureau of Statistics, 2022). In West Sumatra the morbidity rate among the elderly is 20.76%, with 62.62% receiving outpatient care, while (68.66%) self-medicate, 27.32% feel no need for treatment, and 3.63% cite other reasons for not seeking medical care (Indonesian Central Bureau of Statistics, 2022). The rest self-medicate or do not seek treatment (Indonesian Central Bureau of Statistics, 2022). The National Survey also highlights a decline in elderly visits to health facilities in West Sumatra Province, exemplified by the drop in visits to the geriatric polyclinic at Hospital X in Padang City, from 509 in 2019 to 367 in 2020 and 348 in 2021.

Several obstacles have triggered this decline in interest in seeking treatment, including physical limitations, mobility issues, a culture of self-medication, cost difficulties, low family support, inadequate transportation, and long waiting times (Indonesian Central Bureau of Statistics, 2022). This situation is concerning because it delays disease detection and exacerbates health conditions. Therefore, it is crucial to implement health service innovations that can reach the elderly at home [3].

Furthermore, telemedicine services offer a viable solution in many countries. Several studies have shown that telemedicine facilitates access to healthcare services [4], optimizes patient management of geriatric conditions [5], and prevents the worsening of chronic diseases [6]. Therefore, the Indonesian government also promotes telemedicine to bring health services closer to the community, with 36 hospitals in the country already offering these services. However, telemedicine implementation faces challenges such as inadequate platforms [7], weak Information Communication Technology (ICT) infrastructure [8], and lack of user skills [9] among healthcare professionals and patients. Additionally, the absence of specific regulations [10] and poor service coordination between stakeholders [11] further hamper effective implementation. It is estimated that 75% of tele-medicine implementations fail during the operational phase [11].

Moreover, there is a significant lack of in-depth research on the specific needs of the elderly and hospitals, the primary users of telemedicine. The elderly may require support with technology and accessibility, while hospitals need to integrate telemedicine into their existing systems, ensure data security, and comply with regulatory requirements [12]. Successful telemedicine implementation hinges on understanding these user needs, which can be assessed through consultations with key stakeholders [13]. Previous studies have often overlooked the collaborative perspectives of various stakeholders in designing telemedicine services for the elderly [14]. Without a clear understanding of these needs, telemedicine services risk being ineffective and theoretical rather than providing tangible benefits for Indonesia’s elderly population. Therefore, in-depth research on the specific requirements for telemedicine services in geriatric clinics within hospitals is urgently needed [15]. Addressing these challenges through comprehensive studies will provide valuable insights into developing effective telemedicine solutions.

This study aims to develop a framework for geriatric clinic telemedicine [16-18] services by exploring multiple stakeholder perspectives using the Human-Organization-Technology Model (HOT FIT) [19]. The HOT FIT model emphasizes the need for a harmonious alignment between people, organizations, and technology for successful technology implementation. This alignment ensures that telemedicine services meet the needs of users (the elderly), hospital workflows, and technological systems. The study also underscores the importance of external regulations to ensure the effective distribution of telemedicine services. Furthermore, by leveraging Sociotechnical System approaches, which address Health Information Technology (HIT) challenges in complex healthcare systems, the study aims to develop a comprehensive telemedicine service model [20]. This model includes remote consultations, trained home care, structured assessments, regular virtual visits, and multidisciplinary team support [21]. Visual representations of the HOT FIT model can further enhance understanding and serve as valuable tools for implementing telemedicine in geriatric care [22].

## 2 MATERIALS AND METHODS

This study employed a qualitative method with a grounded theory design to explore the telemedicine service needs of a hospital’s geriatric clinic. Additionally, in-depth interviews were conducted using semi-structured guidelines based on the concepts of Human, Organization, Technology-Fit, and Sociotechnical Systems [3]. Informants were purposively selected based on specific criteria until data saturation was reached. Moreover, the informants comprised three groups: elderly patients and their families, health service providers, and related stakeholders. The elderly/family group encompassed various categories: geriatric patients who visit the clinic, elderly patients who go to the hospital but not the geriatric clinic, sick elderly who avoid hospitals, elderly with health complaints who self-treat, and healthy elderly individuals. In addition, family members accompanying these patients were included to ensure the accuracy of the information obtained. On top of that, we included related stakeholders who were managerial staff of the Health Office, as well as the Head of the Community Health Center, who were also included in the sample (Table **1**). The following criteria were used to select informants for the study on a graduated scale: Elderly Patients, Family Members, Health Service Providers, and, Related Stakeholders.

Hospital health service providers, including hospital geriatric consultant specialists, managerial staff, nurses, and information technology (IT) officers, along with related agencies, were included as informants [23]. The elderly informant group comprised various categories: geriatric patients who visit the geriatric clinic, elderly patients who regularly go to the hospital but not to the geriatric clinic, sick elderly who avoid hospitals, elderly with health complaints who self-treat, and healthy elderly individuals. In addition, to ensure the accuracy of the information obtained, elderly informants were accompanied by their families (Table **2**).

The second sample group of health service providers included hospital geriatric consultant specialists who possess extensive knowledge and experience in senior care, particularly in telemedicine services [24]. Their insights are crucial for identifying specific needs and challenges in telemedicine for senior care, as well as for providing valuable perspectives on best practices and implementation strategies. Additionally, these consultants have a broader understanding of the healthcare system and organizational dynamics within hospitals, which is essential for identifying potential barriers to telemedicine implementation and developing effective solutions [25].

Although the small sample size of hospital geriatric consultant specialists may limit the generalizability of their insights, their inclusion can still provide valuable expertise and perspectives that enhance the overall quality and relevance of the project’s findings and recommendations. Additionally, incorporating managerial staff, nurses, and IT officers, along with related stakeholders such as the managerial staff of the Health Office and the Head of the Community Health Center [10, 26-28], ensures a comprehensive understanding of the telemedicine needs and challenges in senior care. Furthermore, the research was conducted in Padang City, West Sumatra, Indonesia, focusing on hospitals with geriatric clinic services, the health office, the Community Health Center, and elderly residents of Padang City. Besides, the interview data underwent analysis involving description of raw data, data reduction, and data categorization according to the HOT FIT approach [19, 29] and the Sociotechnical system [16, 25].

## 3 RESULT AND FINDING

Following in-depth interviews with multiple stakeholders, we present the results of the hospital geriatric clinic telemedicine service needs framework. A total of 18 stakeholder informants were involved, comprising 11 elderly/family informants, 4 hospital service provider informants, and 3 informants from related agencies. Nevertheless, the needs for the geriatric clinic telemedicine service were identified, as shown in Table **3** below [30].

The study delineated three key domains of needs: Technology, Organization, and Human. The geriatric clinic telemedicine service system emerged as comprehensive and dynamic, involving various entities. However, complexity arose due to the services being tailored to vulnerable elderly populations with specific needs. An important discovery was the necessity of considering long-distance drug delivery and its financing regulations, given the limited mobility of the elderly [31]. Community Health Centers and family doctors were identified as pivotal in referrals and facilitating telemedicine visits. Additionally, Posbindu and Integrated Healthcare Centers served as mediums for service socialization. Internal and external regulatory preparations were highlighted as imperative, particularly concerning social security agency health financing schemes and data-sharing agreements between health facilities [32].

Furthermore, the resulting hospital geriatric clinic telemedicine service model requirements framework comprehensively considered various aspects, aiming to guide a robust implementation framework in the future. These findings underscore the importance of addressing technological, organizational, and human factors in implementing telemedicine services for the elderly, particularly focusing on their specific needs and the complex nature of delivering care to this population [33].Therefore, the resulting hospital geriatric clinic telemedicine service model requirements framework comprehensively considers various aspects to guide its implementation in the future [34].

## 4 DISCUSSION

Telemedicine geriatric clinics present a contemporary alternative to traditional outpatient visits, delivering remote geriatric clinic services through communication technology. These clinics offer advanced care while upholding the principle of social distancing, which is particularly advantageous for ensuring safety in diverse healthcare settings.

### 4.1 Technology Aspect Needs

#### 4.1.1 Human-Computer Interface

The Human-computer Interface (HCI) in telemedicine geriatric clinic services holds significance in delivering effective and efficient remote services to elderly patients [35]. Research underscores the necessity of devices such as computers or laptops for service providers like hospitals, health centers, and family doctors [13], in providing telemedicine services. A suitable application platform is vital, with an Android-based mobile application catering to elderly users and families alongside a comprehensive and integrated platform for service providers [36]. The application should prioritize high audio-visual quality for teleconferencing interfaces, facilitating clear visual examination and detailed hearing of patient complaints. Acceptance and utilization of telemedicine technology are influenced by users’ perceptions of its usefulness and ease [37], thus highlighting the importance of trust among the elderly, service providers, and related agencies in the technology’s efficacy [14]. Moreover, designing software components that cater to the user population is essential. The utilization of HCI in telemedicine geriatric clinics holds promise for enhancing system quality and medical services provided *via* telemedicine, signifying a positive shift in the paradigm of health services through telemedicine [35].

Furthermore, to ensure the successful adoption and implementation of telemedicine services for the elderly, several key strategies should be considered. Firstly, conducting usability testing with elderly users can identify and address potential adoption barriers, ensuring that the telemedicine platform is intuitive and user-friendly [38]. Secondly, involving elderly users in the design process can yield valuable feedback and insights into their preferences and needs, fostering a more user-centered approach. Emphasizing the importance of trust in the usefulness of telemedicine technology among elderly users, service providers, and related agencies is therefore crucial for acceptance and sustained use [39]. Additionally, integrating insights from interviews or surveys capturing stakeholders’ perceptions of telemedicine can illuminate how these perceptions influence acceptance and usage [29]. In addition, including a detailed economic analysis section evaluating the cost-effectiveness of the telemedicine system is imperative, considering factors such as initial investment, operational costs, and potential cost savings compared to traditional healthcare delivery models. Highlighting the economic benefits of telemedicine can bolster the case for its implementation and long-term sustainability.

#### 4.1.2 Infrastructure

Telemedicine geriatric clinic services necessitate application software integrated with Electronic Health Records (EHR), data management functions, data transfer capabilities, schedule setting, waiting room features, and two-way audio-video functions. An easily downloadable mobile application is essential for elderly/family users. Integrating the geriatric clinic telemedicine application with the EHR enables real-time updates of patient medical information and enhances system interoperability. Equally, an organized data management function ensures a seamless experience in managing elderly health information. A platform infrastructure facilitating data transfer between telemedicine applications in hospitals, health centers, and family doctors enhances service quality. Appointment scheduling and waiting room features within the app streamline access to schedule information for elderly patients and optimize waiting times. A robust WiFi network forms a critical foundation in the system infrastructure, ensuring high-quality telemedicine services that are easily accessible to the elderly population [40]. Hence, prioritizing accessibility through easy-to-use mobile apps is particularly crucial for older adults lacking experience in technology usage [41].

The study’s approach to telemedicine infrastructure and its consideration of the needs of different stakeholders demonstrate a commitment to sustainability and scalability, even in Indonesia’s less urbanized or resource-constrained areas [11]. Likewise, by addressing these key aspects, the study lays the foundation for a telemedicine framework capable of adapting to varying conditions and effectively meeting the healthcare needs of the elderly population [42].

#### 4.1.3 Workflow and Communication

Ensuring smooth and efficient delivery of geriatric telemedicine clinic services and constructing a high-quality system for the elderly [43] hinges on good workflow and communication. Research findings on workflow and communication needs in the technological aspect of telemedicine geriatric clinic services reveal the requirement for three distinct login/sign-in flows for elderly users accessing the telemedicine geriatric clinic service application at the hospital. These flows cater to elderly citizens logging in from home, those logging in through the Community Health Center doctor as an intermediary, and family doctors with access to services [44]. Features for data gathering during registration, including self-screening, aid in collecting initial information about the patient’s health status and assist in prioritizing treatment by doctors.

Another crucial aspect is the inclusion of payment features within the application to facilitate easy and secure payment for services by the elderly and their families, obviating the need to visit the hospital directly [45]. This feature is especially beneficial for elderly patients unable to travel to the pharmacy to collect prescriptions, as they can utilize the e-prescription feature in the pharmacy department. The dimensions of workflow and communication in the technological aspects of telemedicine geriatric clinic services rely on the clarity of features and functioning of the system to promote cohesive communication [8]. The Sociotechnical System Model Centered On The User theory by Sittig and Singh emphasizes that communication patterns and workflows are shaped not only by the study design but also by the organizational structure, pre-existing workflow patterns, and existing clinic protocols [39, 46, 47].

#### 4.1.4 Clinical Content

Clinical content encompasses all data, information, and knowledge stored in and related to the system. Findings regarding information needs in the technological aspect of this study underscored essential elements requisite in the technological infrastructure of hospital telemedicine services.

##### 4.1.4.1 Patient Information, Simple Screening Data, and Patient Complaints

Patient information, simple screening data, and patient complaints form pivotal components. The collection of patient information data begins at the time of registration/patient registration in the telemedicine application in a Store-and-Forward or Asynchronous manner [26, 48, 49]. Subsequently, the necessity arises for patient clinical data in the form of screening data and patient complaints, encompassing disease symptoms or previous treatment history. The requirement for simple screening data, conducted independently by the patient/family or through the health center or family doctor, alongside initial complaints, is imperative [7, 40, 50]. Such information aids doctors in comprehending a patient’s condition before consultation.

##### 4.14.2 Data Integration with Medical Records for Old and New Patients

Data integration with the medical record system is crucial as it enables doctors to access the patient’s complete medical history, encompassing old and new patients. This ensures that treatment can be tailored to address both previous and current conditions. Despite numerous methods aimed at overcoming the challenges of EHR interoperability, including the establishment of technical standards by various international organizations such as the International Standards Organization (Health Level 7 (HL7) standards, these efforts have yet to yield a collective impact [21, 51, 52]. Therefore, addressing limitations and challenges, such as linking the telemedicine geriatric clinic service platform with various EHRs in the hospital, as well as fostering interoperability between different EHRs, remains imperative.

##### 4.1.4.3 Geriatric Sub-specialist Doctor Data and Clinic Service Schedule

Geriatric sub-specialist doctor data and clinic service schedules enable patients to identify which specialists are providing services and adjust their schedules accordingly.

##### 4.1.4.4 Clinical Content Delivered through Chatbots/AI

Clinical content deliverable through chatbots/AI serves as an assistance tool to provide basic information and initial assistance to patients. However, the use of this technology requires consideration of data protection and patient ethics, particularly in a healthcare environment.

##### 4.1.4.5 Data Safety

Data safety is a paramount concern in the technological aspect of geriatric telemedicine clinic services. A key finding regarding clinical content underscores the need for data security to protect patient data. Basic information security principles, such as confidentiality, integrity, and availability, are often associated with the Confidentiality, Integrity, Availability (CIA) framework [53]. This framework ensures that all data transferred between the telemedicine platform and EHRs is encrypted and securely transferred, maintaining patient data confidentiality and protection from unauthorized access during transmission [38]. Therefore, implementing strict access controls and authentication mechanisms ensures that only authorized personnel can access patient data, minimizing the risk of unauthorized access and data breaches. Additionally, by minimizing the amount of data collected and anonymizing data when possible, the framework can reduce the risk of exposing sensitive information, particularly in geriatric medical data, which may contain sensitive personal information [54]. Overall, implementing these mechanisms ensures compliance with international data protection standards and addresses potential breaches, thereby safeguarding the confidentiality and security of geriatric medical data within telemedicine services. [55].

##### 4.1.4.6 Elderly Health Information

Clear information on caring for the elderly at home, along with elderly health information and advice and follow-up from doctors [56], are essential for elderly patients and their families. This information aids in long-term care, necessitating a doctor’s note page in the application where doctors can input health information needed by the elderly and families at home, ensuring easy accessibility [17, 19, 38, 39].

### 4.2 Needs on the Human Aspect

Extensive human resource needs were identified in hospital telemedicine geriatric clinic services. In the service user dimension, the study revealed diverse conditions among the elderly targeted by the service, ranging from independent to those with various limitations. Consequently, an individualized assessment by a geriatrician is necessary to determine the eligibility of the elderly for telemedicine use and consider adjusting the focus of the consultation clinic to meet achievable goals [18]. From the provider side, geriatric clinics necessitate multidisciplinary collaboration involving geriatricians, trained general practitioners, nurses, and IT and management personnel. Effective coordination between teams is crucial for telemedicine services to adequately address the multidimensional needs of the elderly [10].

Furthermore, integrating hospital geriatric [27, 31, 57] clinic telemedicine services with community health centers and family doctors proves crucial in bringing closer access to the elderly. Community Health Centers and family doctors, serving as primary services, can act as referrals and facilitators in providing telemedicine geriatric clinic services for older people who face difficulties reaching hospitals or prefer not to go to them or for those elderly individuals who live alone and lack access. Meanwhile, the involvement of Integrated Healthcare Center Lansia and Posbindu is limited to socialization and education about the telemedicine program at the geriatric clinic to the elderly and their families in the community. Overall, close collaboration between various stakeholders is vital to optimizing human resources and enhancing the accessibility and quality of geriatric clinic health services through telemedicine [48].

### 4.3 Needs on the Organizational Aspect

This aspect encompasses various elements that must synergize with the adopted technology to establish conducive conditions for the successful implementation of Telemedicine.

#### 4.3.1 Ecosystem

The research findings regarding the needs of the hospital telemedicine geriatric clinic service ecosystem underscore the importance of collaboration by engaging diverse elements in providing geriatric clinic services *via* telemedicine. This collaboration aims to foster a closely integrated innovation framework. Congruently, the necessity for collaboration between geriatric clinic services in hospitals and Community Health Centers and family doctors illustrates a comprehensive service ecosystem, extending beyond the confines of the hospital organization to encompass the broader community. This approach aims to establish an extensive tele-medicine geriatric clinic service innovation network within the community.

Apart from that, the study’s primary discovery regarding the requirement for a collaborative framework involving hospitals, health centers, and family physicians within the geriatric telemedicine ecosystem is groundbreaking. It contributes to the development of the collaborative innovation ecosystem concept [58]. The proposed framework has the potential to enhance access to geriatric health services in the community through the facilitative roles of Community Health Centers and family doctors. However, implementation challenges may surface concerning the readiness of IT infrastructure at Community Health Centers and family doctors, as well as potential resistance to change among medical personnel. [59]. Consequently, comprehensive regulations, socialization efforts, and training initiatives are imperative before implementing the framework. In comparison to prior studies, this research enriches the understanding of a more comprehensive digital geriatric [31, 33, 57] healthcare collaboration model by incorporating elements of Community Health Centers and family doctors.

#### 4.3.2 Workflow and Communication

Our study identifies three sets of telemedicine services for hospital geriatric clinics, aiming to provide comprehensive care for elderly patients. These services allow elderly patients to access care directly from home, through health centers, or *via* family doctors. Each approach accommodates different patient situations and fosters effective communication between patients and medical personnel [60]. The proposed model for hospital telemedicine geriatric clinics, which involves collaboration with primary healthcare facilities, represents a significant step towards developing Indonesia’s integrated digital geriatric healthcare system. This model aims to improve elderly accessibility to hospitals by leveraging primary healthcare facilities as facilitators. However, challenges such as IT infrastructure readiness, funding policies, and HR competencies may impede implementation. Further studies are necessary to evaluate the readiness for implementing this model. Overall, this service flow model has the potential to create integrated and collaborative digital geriatric health services in Indonesia, particularly in Padang City.

#### 4.3.3 Internal Organization Features

Establishing internal hospital policies and procedures related to information technology use, service standards, and ethical professionalism is crucial for a safe and quality geriatric telemedicine clinic program [61]. Hospitals must integrate ethical and professional principles into their internal policies and procedures when implementing telemedicine geriatric clinic services [55]. For instance, obtaining informed consent before video consultations ensures that patients agree and are prepared for the telemedicine sessions. Also, privacy and confidentiality of patient data should be maintained through data encryption and strict access controls. Additionally, respecting patient autonomy is vital, allowing them to choose telemedicine services or opt for face-to-face consultations as needed [62]. By addressing these foundational aspects, hospitals can ensure the successful and ethical implementation of telemedicine services, ultimately enhancing the quality of care provided to elderly patients. Hospitals often face a lack of staff support when implementing new policies. In addition, to address this, they must conduct comprehensive socialization efforts through seminars and training sessions. Overcoming internal bureaucratic obstacles requires multidisciplinary teamwork to ensure smooth policy implementation.

#### 4.3.4 External Regulation

Our findings indicate that external regulations are essential to support hospital geriatric telemedicine services. Formulating a Minister of Health Regulation on standard tariffs for geriatric telemedicine services covered by the Social Security Agency on health is crucial to comply with the human-centered design principle from sociotechnical system theory. Despite the issuance of Minister of Health Regulation No. 3 of 2023 concerning Health Service Tariff Standards, telemedicine services in hospitals are still not included in the standard tariffs covered by the Social Security Agency on health. Moreover, to effectively organize telemedicine geriatric clinic services, service providers need clear regulations, such as Mayor’s Instructions or Regional Regulations, to govern cooperation between hospitals and first-level health facilities like Community Health Centers and family doctors. Establishing a clear legal basis is essential. Additionally, the Memorandum of Understanding (MOU) between the hospital and the Social Security Agency on health plays a crucial role in implementing geriatric telemedicine services. This MOU should cover not only service rates but also the referral process of elderly patients from primary healthcare providers directly to type B hospitals or type A hospitals, given the limited geriatric clinic services available in type C hospitals in Padang City.

Hospitals need MOUs with the City Health Office and Community Health Centers to clarify the use of geriatric telemedicine clinic applications. MOUs with family doctors are also essential to integrate primary and secondary care effectively. Additionally, agreements for data sharing between Community Health Centers, family doctors, and hospitals are necessary to ensure continuity of service and maintain intact patient medical information. From a theoretical perspective, our findings align with the HOT Fit model theory [1, 19, 51]. External regulation involves fitting organizational aspects to the local context and situation. Such regulations ensure that telemedicine services meet the needs of users, align with hospital work processes, and integrate with the technology systems used. For instance, a Minister of Health Regulation is necessary to standardize telemedicine technology for health services in Indonesia [63]. Equally, local government regulations must adjust to the local context, and MOUs between hospitals, the Social Security Agency on Health, and DHOs are needed to align telemedicine services in geriatric clinics with health service and funding mechanisms. Just as MOUs with Community Health Centers and family doctors help integrate these services into the broader health system [28, 34, 60]. Therefore, external regulations are crucial to achieving compatibility among human, organizational, and technological elements.

Intriguingly, the Mayor’s Instruction and local regulations on telemedicine cooperation between health facilities should reflect the principle of contingency, fitting the local situation and context. Similarly, hospital MOUs with the Social Security Agency on health and DHOs should incorporate the principle of joint optimization from sociotechnical theory, integrating technical and work systems. Taken together, comprehensive regulations based on these principles can support the effective and high-quality implementation of telemedicine services [1, 13, 64].

#### 4.3.5 Financial Source Feature

Our findings indicate that payments for telemedicine geriatric clinic services need coverage by both the Social Security Agency on Health and the patients [65]. To reduce medical costs for the elderly, the Social Security Agency on Health should approve claims for telemedicine services, providing hospitals with financial certainty [66]. For elderly individuals not covered by social security agencies, an independent payment scheme is necessary, as not all can afford the full cost of services. Hospitals should consider offering tiered or subsidized payment options for these patients to ensure they can still access telemedicine services. With flexible financing schemes from the Social Security agency and hospitals, telemedicine geriatric clinic services can be made accessible to all elderly individuals without exception.

Conducting a cost-effectiveness analysis is crucial for determining the most efficient methods of delivering telemedicine geriatric clinic services. This analysis can compare the costs and outcomes of various service delivery models, identifying areas where costs can be reduced without compromising quality. Thus, implementing tariff schemes and tiered or subsidized payments for elderly individuals not covered by social security can ensure that all elderly patients have access to telemedicine services without financial hardship. These schemes can be designed based on income levels or other relevant criteria, ensuring those who need the most support receive it. Flexible financing plans from social security agencies and hospitals, including negotiated payment terms and payment plans for patients who cannot afford the full cost upfront, can help maintain the financial sustainability of telemedicine services. Additionally, public-private partnerships can mobilize additional resources, with private sector partners contributing expertise and funding, thus reducing the financial burden on hospitals and healthcare providers.

Likewise, by distributing the financial responsibility for telemedicine services between the Social Security agency and the patients, the model alleviates the financial burden on any single party while ensuring hospitals receive necessary financial support. Implementing flexible financing plans from both social security agencies and hospitals can sustain the financial viability of telemedicine services. These plans might involve negotiating payment terms with social security agencies and offering payment options for patients who cannot pay the full cost upfront. The model also emphasizes the importance of conducting cost-effectiveness analyses to identify the most efficient ways to deliver telemedicine services. This approach ensures that costs are minimized without compromising quality, thereby supporting the financial sustainability of telemedicine services.

The findings align with the concept of Universal Health Coverage in Indonesia, which emphasizes equitable access to health services regardless of socioeconomic status. Implementing a flexible financing plan involving both the Social Security Agency and hospitals ensures that everyone can access telemedicine geriatric clinic services, thereby optimizing universal health coverage. This study’s results have positive implications for expanding elderly access to health services in the future.

## 5 RESEARCH LIMITATIONS

While this study explored the development of a geriatric clinic telemedicine service framework with a diverse range of stakeholders, a larger number of informants would be necessary to further deepen and expand the framework [20, 52]. We cannot yet demonstrate whether using this framework will lead to successful implementation in hospitals. However, it offers a valuable tool for the rapid prototyping and iteration of telemedicine systems, facilitating the creation of collaborative and innovative geriatric telemedicine service programs in Padang City hospitals.

## CONCLUSION

This telemedicine geriatric clinic service needs framework provides a comprehensive visualization of service requirements, encompassing technological, human, and organizational aspects. Hospital service providers in Padang City must consider these ten key dimensions to successfully implement telemedicine geriatric clinic services. The framework serves as a planning tool to evaluate and iterate on unmet aspects, ensuring all relevant factors are addressed [3, 43, 67]. Moreover, by identifying potential obstacles early and addressing key needs proactively, organizations can avoid issues that might hinder the success of geriatric telemedicine programs, leading to more successful implementations [13, 68]. In conclusion, this study provides a comprehensive framework for implementing telemedicine geriatric clinic services in Padang City, addressing technological, human, and organizational aspects. By identifying ten key dimensions, the framework enables hospital providers to proactively address potential obstacles and ensure successful implementation, ultimately enhancing elderly patients’ access to quality healthcare services.

Future research should involve a larger number of participants and include other regional public and private hospitals in Indonesia to validate the generalizability of the geriatric clinic telemedicine service needs framework [69]. IT studies focused on telemedicine system development and longitudinal studies evaluating the implementation and impact on elderly health outcomes are recommended [54, 64, 70]. Additionally, an in-depth study is necessary to assess the readiness of the regulatory model and social security agency financing schemes to support telemedicine services. Besides, involving a network of relevant stakeholders in policy development for implementing the hospital geriatric clinic telemedicine service model in Indonesia is also advisable. We, therefore, recommend that hospitals in Padang City adopt the identified framework to improve the implementation of telemedicine geriatric services, focusing on early-stage consideration of key dimensions. The main limitation of this study is the relatively small sample size, which may affect the generalizability of the findings. Future studies should include a larger and more diverse group of participants from various regions in Indonesia, along with IT and longitudinal studies, to evaluate the framework’s impact on elderly health outcomes and the readiness of regulatory models and financing schemes.

## Figures and Tables

**Table 1:** Description of elderly patients.

**Population**	**Sample**	**Age**	**Gender**	**Education**
75. 800	1	73 Th	Female	Primary school
	2	68 Th	Female	High school
	3	62 Th	Female	Division two
	4	61 Th	Female	High school
	5	69 Th	Female	Bachelor
	6	72 Th	Male	Primary school
	7	69 Th	Female	Primary school
	8	69 Th	Female	Primary school
	9	61Th	Female	High school
	10	67 Th	Female	Primary school
	11	72 Th	Female	Primary school

**Source:** Primary Data (2023).

**Table 2:** Characteristics of respondents.

**Respondents' Characteristics**	**(%)**
1	3
Education	
Primary school	54.54
High School	27.27
Division 2	9.09
Bachelor	9.09
Knowledge about Telemedicine Geriatric Clinic Services	
Do Not Know	100
Ability to use a Smartphone	
Know	36.36
Do Not Know	63.63
Willingness to use the service	
Willing to Use	81.81
No (Face-to-Face)	18.18

**Table 3:** Framework for geriatric telemedicine clinic service model needs in hospital.

**Construct Framework of HOT FIT Model and Sociotechnical System**	**Needs**
**1. Technology**	
• **System Quality**	
• Human-Computer Interface	• Computer, laptop, and smartphone (cellphone). • Mobile Android application platform for elderly/family, comprehensive and integrated application for service providers. • Application with high audio-video quality for teleconferencing so that it can support objective and subjective examinations.
• Infrastructure	• Application software integrated with Electronic Health Record (EHR) (interoperability). • Data management function (medical record), data transfer, schedule setting, waiting room, two-way audio-video function. • Powerful wifi network. • Downloadable mobile application software for easy use.
• Workflow and communication	• Three elderly flows to be able to log in to the hospital telemedicine geriatric clinic service application, namely: elderly patients themselves log in from home, community health center through community health center doctors log in, and family doctors log in because for technological needs, 3 accounts are needed to log in. • Data gathering requirements for registers, including self-screening. • Payment features. • Video call. • E-prescription. • Drug delivery.
• **Information Quality**	
• Clinical Content	• Simple screening data, patient information, and complaints. • Integration with medical records for existing and new patients. • Data on sub-specialist doctors and service schedules at polyclinics. • Clinical content can be delivered by chatbot/AI. • Has a data security system (Data Safety). • Information on how to take medication at home, information on the health of the elderly, follow-up, and doctor's advice (doctor’s notes for elderly care at home).
**2. Human**	• The importance of telemedicine geriatric clinic services provided in hospitals is more aimed at educating elderly patients and their families about elderly diseases and how to care for them at home, given the habit of elderly people in Padang city only visit hospitals for outpatient treatment if they feel sick, but if they feel less sick, they prefer to self-medicate or ignore it. **Elderly/Family Users:** • Elderly people in good condition, with mild cases of illness, who are able to do telemedicine on their own and are educated. • Elderly with certain conditions such as, immobilization, comorbidities, families who cannot take them or, elderly who do not want to be taken to the hospital, and elderly who do not have families. • The elderly continue to visit the health facility every month. • The use of the service is more for the family of the elderly because the elderly themselves who use the telemedicine service will experience difficulties. **System users from the service provider** • Consultant physician in geriatrics. • General practitioners who have received training in geriatrics. • Nurse as operator. • IT personnel as operators. • Service director management and digitization. **Relevant agencies that act as service providers in collaboration with hospitals:** • Health centers consisting of health center doctors, nurses, and other health workers when needed. • Other relevant stakeholders do not function as service providers, but as providers of information about telemedicine geriatric clinic services in collaboration with Community Health Center. • Elderly Integrated Healthcare Center officer. • Posbindu Officer.
**3. Organization**	
• Ecosystem	• Ecosystem area; geriatric clinic integrated with medical record installation, pharmacy (interoperability). • Community health center corner for referrals. • Service functions include consultation and education, drug service and delivery, follow-up for patients who have visited the polyclinic or been treated on referral, and fast track.
• Workflow and communication	It takes 3 (three) service flows of the hospital telemedicine geriatric clinic, namely: • Elderly patient from home: Elderly/family log-in to app (Profile account) → registration and data collection → Family self-screening → Communication with operator/nurse (tele-triage) → Appointment meeting (online meeting) → Payment → Video consultation → Pharmacy prescription → Drug delivery → completed. • Elderly patients from the health center: General practitioner logs in to the application → Registration and input of patient data report → Communication with operator/nurse → Appointment → Video consultation → Completed. • Elderly patients from family doctors: Family doctor logs in to the application → Registration and input of patient data report → Communication with operator/nurse → Appointment → Video consultation → Completed.
• Internal Organization Feature	• Provisions on the use of IT. • Provisions on the implementation of telemedicine services include doctor’s schedule, length of service, service during working hours and outside working hours, doctor’s flexibility, and service rates.
• External Regulation	External regulations are needed, among others: • Minister of Health Regulation on geriatric clinic telemedicine services paid by Social Security Agency on Health. • Mayor’s Instruction or Regulation from Local Government. • Sk Dinas Kesehatan for health centers that collaborate with hospitals to provide hospital telemedicine geriatric clinic services. • Memorandum of Understanding (MOU) between hospital and social security agency on health. • MOU of the hospital with the city health office and community health center on the use of the hospital's geriatric telemedicine clinic plenary service application. • Hospital MOU with family doctors. • Agreement for data sharing between health centers, family doctors, and hospitals.
• Financial Source Feature	• Financing sources for telemedicine services by the elderly are expected to be from the Social Security Agency on health or independent/self-funded. • The source of financing for service provision by hospitals is from the hospital's own budget by adjusting the hospital's needs, or by utilizing the telemedicine platform provided by the Indonesian Ministry of Health.

**Note:** The results of research findings from multiple perspectives constructed from the HOT FIT Model theory [30].

## Data Availability

The data and supportive information are available within the article.

## LIST OF ABBREVIATIONS

CIA: Confidentiality, Integrity, Availability
DHOs: District Health Offices
EHR: Electronic Health Record
HCI: Human-computer interface
HIT: Health Information Technology
HOT FIT: Human-Organization-Technology
ICT: Information and Communication Technology
IT: Information Technology
MOU: Memorandum of Understanding

## STANDARDS OF REPORTING

COREQ guidelines were followed.
